# Evaluation of 5 Commercially Available Zika Virus Immunoassays

**DOI:** 10.3201/eid2309.162043

**Published:** 2017-09

**Authors:** David Safronetz, Angela Sloan, Derek R. Stein, Emelissa Mendoza, Nicole Barairo, Charlene Ranadheera, Leanne Scharikow, Kimberly Holloway, Alyssia Robinson, Maya Traykova-Andonova, Kai Makowski, Kristina Dimitrova, Elizabeth Giles, Joanne Hiebert, Rhonda Mogk, Sharla Beddome, Michael Drebot

**Affiliations:** Public Health Agency of Canada, Winnipeg, Manitoba, Canada

**Keywords:** Zika virus, viruses, immunoassays, ELISA, IgM-capture ELISA, MAC-ELISA, IgM, evaluation, sensitivity, specificity, vector-borne infections, zoonoses, Canada

## Abstract

Because of the global spread of Zika virus, accurate and high-throughput diagnostic immunoassays are needed. We compared the sensitivity and specificity of 5 commercially available Zika virus serologic assays to the recommended protocol of Zika virus IgM-capture ELISA and plaque-reduction neutralization tests. Most commercial immunoassays showed low sensitivity, which can be increased.

Zika virus is a mosquito-borne member of the family *Flaviviridae*, genus *Flavivirus*, that was originally discovered in 1947 in Uganda (*1*). For several decades, Zika virus seemed to be geographically restricted to equatorial Africa with a few documented incursions into Asia (*2*,*3*). Although several studies demonstrated serologic evidence of human exposures to Zika virus across Africa, it was believed that this virus was not a major public health threat. However, in 2007, the epidemic potential of Zika virus became apparent when it was identified as the causative agent of an outbreak in Yap State, Federated States of Micronesia, which consisted of 49 confirmed cases, 59 probable infections, and dozens more suspected cases (*4*,*5*). Since 2007, several epidemics have occurred across the Pacific Ocean Region, including an outbreak in 2013–2014 with thousands of confirmed cases in French Polynesia (*6*).

In 2015, the first cases of Zika virus infection were confirmed in Brazil, which indicated the beginning of the largest outbreak recorded with autochthonous vectorborne transmission documented in >65 countries across the Americas (*2*,*3*,*7*). Although it is still widely believed that most Zika virus infections in humans are asymptomatic or mild with self-limiting clinical manifestations, it is now documented that Zika virus infections can lead to major complications and long-term sequelae, including congenital birth defects, neurologic disorders, and prolonged risk for sexual transmission of this virus (*3*,*8*).

Before 2007, only 14 laboratory-confirmed cases of Zika virus infection had been documented worldwide. Thus, it is not surprising that diagnostics for Zika virus were conducted only in specialized arbovirus reference laboratories (*2*). During the outbreak on Yap Island, samples were sent to the Arbovirus Diagnostic Laboratory, Centers for Disease Control and Prevention (Fort Collins, CO, USA), where molecular and serologic assays were quickly developed for confirmatory testing (*5*). Many of these in-house methods developed in 2007, including real-time molecular assays, an IgM-capture ELISA (MAC-ELISA), and a plaque reduction neutralization test (PRNT), have been used during the current outbreak. However, the magnitude of the outbreak, combined with the in-house production of key reagents involved in diagnostics of Zika virus infection, has taxed the few reference laboratories capable of producing, standardizing, and distributing such materials. Therefore, application and evaluation of sensitive and specific diagnostic assays, particularly those that can be used in frontline laboratories, has become a top public health priority.

Several laboratories and commercial vendors have developed and evaluated molecular assays for rapid identification of Zika virus RNA, and, in some instances, other clinically relevant arboviruses, such as dengue virus (DENV) and chikungunya virus, in acute-phase clinical specimens (*9*). However, high-throughput commercially produced immunoassays have proven to be more challenging because of strong serologic cross-reactivity of closely related flaviviruses, such as DENV. We compared the sensitivity and specificity of 5 commercially available Zika virus serologic assays to the recommended protocols of Zika virus MAC-ELISA and PRNT.

## The Study

Samples were submitted to the National Microbiology Laboratory of the Public Health Agency of Canada (Winnipeg, Manitoba, Canada) for arbovirus diagnostic testing. All samples were obtained from Canadian travelers who visited areas with known Zika virus transmission and consulted their physicians after symptoms consistent with Zika virus infection developed upon return.

We obtained deidentified samples from 75 patients. Thirty samples were from patients with serologically confirmed Zika virus infections; 10 from patients with confirmed Zika virus infections identified by 2-target real-time reverse transcription PCR (RT-PCR); 10 from patients with suspected Zika virus infections, which were subsequently identified as DENV infections; and 25 acute-phase samples from flavivirus-negative persons tested by Zika virus RT-PCR and MAC-ELISA. Primary Zika virus diagnostic testing for all samples was conducted by using an in-house CDC-based MAC-ELISA and subsequent confirmation of Zika virus infection by cross-PRNTs for Zika virus and DENV, or molecular assays as described (*5*).

We evaluated 5 Zika virus immunoassays in this study. We tested a conventional IgM ELISA (EI 2668-9601 M; Euroimmun AG, Luebeck, Germany) and 3 MAC-ELISAs: Zika Virus Detect (InBios International Inc., Seattle, WA, USA); Ab213327 (Abcam, Cambridge, UK); and NovaLisa ZVM0790 (Novatec Inc., Baltimore, MD, USA). On the basis of preliminary testing, we also tested the Euroimmun IgM ELISA in parallel with the Euroimmun conventional Zika virus IgG ELISA (EI 2668-9601 G). Both Euroimmun assays use recombinant Zika virus nonstructural protein 1 as the antigen; the InBios Zika Virus Detect uses a recombinant Zika virus envelope glycoprotein as the positive antigen, an unspecified cross-reactive control, and reference cell antigens; and the Novatec and Abcam ELISAs use an unspecified Zika virus antigen.

Most tests evaluated provided algorithms that resulted in positive, negative, or equivocal results. However, the InBios kits account for antigenics associated with secondary flavivirus infections and reports results as Zika virus positive, possible Zika virus positive, or presumptive other flavivirus positive or negative on the basis of calculations of optical density ratios obtained from a sample with the 3 different antigens. Two independent laboratory technicians blindly evaluated the 5 assays by using the panel outlined, according to the manufacturer’s instructions. Comparisons and performance calculations were conducted by the Quality Control Office of the National Microbiology Laboratory.

The assays generally showed reproducible results during independent evaluations, although specificity and sensitivity of each varied (Table 1). The Euroimmun IgM and IgG ELISAs and the Abcam IgM ELISA showed a specificity of 100% for negative specimens with similar results (>90%) for confirmed DENV-positive samples (Table 2). The NovaTec ELISA showed a specificity of 66% for negative specimens and 70% for DENV-positive specimens. Although the InBios Zika Detect ELISA showed similar specificity results for flavivirus-seronegative specimens, it showed decreased specificity for DENV-positive samples. This assay incorrectly identified 40% of these samples as Zika virus IgM positive and 40% as possible Zika virus positive.

**Table 1:** Results of in-house and commercially available Zika virus immunoassays*

Sample collection dpo	In-house Zika virus diagnostic results			DENV PRNT titer	Commercial Zika virus serologic assays results				
	RT-PCR	MAC-ELISA	PRNT titer		Euroimmun IgM	Euroimmun IgG	Novatec IgM	Abcam IgM	InBios IgM
12	ND	Pos	>40	Neg	Pos	Pos	Pos	Pos	Pos
9	ND	Pos	>40	Neg	**Neg**	**Neg**	**Neg**	**Neg**	Pos
4	ND	Pos	>40	Neg	**Neg**	**Neg**	**Neg**	Pos	Pos
27	ND	Pos	>40	Neg	Pos	Pos	Pos	Pos	Pos
39	ND	Pos	>40	Neg	**Neg**	Pos	Pos	Pos	Pos
11	ND	Pos	>40	Neg	Pos	Pos	Pos	Pos	Pos
109	ND	Pos	1,280	20	**Neg**	Pos	**Neg**	**Neg**	Pos
49	ND	Pos	>40	Neg	**Neg**	Pos	Pos	Pos	Pos
Unknown	ND	Pos	>40	Neg	**Neg**	**Neg**	**Neg**	**Neg**	Pos
4	ND	Pos	>40	Neg	Pos	**Neg**	Pos	Pos	Pos
7	ND	Pos	>40	Neg	**Neg**	Pos	**Neg**	**Neg**	Pos
46	ND	Pos	>40	Neg	**Neg**	Pos	Pos	Pos	Pos
Unknown	ND	Pos	>40	Neg	**Neg**	Pos	**Neg**	**Neg**	Pos
Unknown	ND	Pos	>40	Neg	**Neg**	Pos	Eq	Pos	Pos
118	ND	Pos	>40	Neg	**Neg**	Pos	**Neg**	**Neg**	Pos
57	ND	Pos	>40	Neg	Pos	**Neg**	Pos	Pos	Pos
66	ND	Pos	>40	Neg	**Neg**	Pos	**Neg**	Eq	Pos
43	ND	Pos	40	Neg	**Neg**	Pos	Eq	Pos	Pos
2	ND	Pos	>40	Neg	**Neg**	**Neg**	Pos	**Neg**	Pos
41	ND	Pos	>40	Neg	Pos	Pos	**Neg**	**Neg**	Pos
5	ND	Pos	>40	Neg	**Neg**	**Neg**	**Neg**	**Neg**	Pos
38	ND	Pos	>40	Neg	**Neg**	Pos	**Neg**	**Neg**	PZ
4	ND	Pos	>80	Neg	Pos	**Neg**	**Neg**	Pos	Pos
6	ND	Pos	>80	Neg	Pos	Pos	**Neg**	**Neg**	Pos
2	ND	Pos	>80	Neg	Neg	Pos	**Neg**	Eq	Pos
12	ND	Pos	40	Neg	Pos	**Neg**	Pos	Pos	Pos
Unknown	ND	Pos	>80	Neg	Pos	**Neg**	Pos	Pos	Pos
28	ND	Pos	>80	Neg	Pos	Pos	**Neg**	**Neg**	Pos
75	ND	Pos	>80	20	**Neg**	Pos	**Neg**	**Neg**	Pos
68	ND	Pos	>40	Neg	**Neg**	Pos	**Neg**	**Neg**	Pos
9	ND	**Pos**	Neg	>40	Neg	Neg	Neg	Neg	PZ
7	ND	**Pos**	Neg	>40	Neg	Neg	Neg	Neg	PZ
31	ND	**Pos**	Neg	>40	Neg	Neg	Neg	Neg	PZ
6	ND	**Pos**	Neg	>40	**Pos**	**Pos**	Neg	Neg	Pos
20	ND	**Pos**	Neg	>40	Neg	Neg	**Pos**	**Pos**	PZ
36	ND	**Pos**	Neg	>80	Neg	Neg	Neg	Neg	OF
Unknown	ND	**Pos**	320	>5,120	Neg	Neg	Neg	Neg	Pos
Unknown	ND	**Pos**	Neg	40	Neg	Neg	**Pos**	Neg	Neg
3	ND	**Pos**	Neg	>80	Neg	Neg	Eq	Neg	Pos
Unknown	ND	**Pos**	Neg	>640	Neg	Neg	Neg	Neg	Pos

*Bold indicates false-positive/false-negative results. Underlining indicates inconclusive results that required further testing. DENV, dengue virus; dpo, days postsymptom onset; Eq, equivalent; MAC-ELISA, IgM-capture ELISA; ND, not done; Neg, negative; OF, other flavivirus; Pos, positive; PRNT, plaque reduction neutralization test; PZ, possible Zika virus; RT-PCR, reverse transcription PCR.

**Table 2:** Evaluation of commercial Zika virus serologic assays on Zika virus–negative samples*

Sample collection dpo	Zika virus RT-PCR	Zika virus MAC-ELISA	Euroimmun IgM	Euroimmun IgG	Novatec IgM	Abcam IgM	InBios IgM
Unknown	Neg	Neg	Neg	Neg	Neg	Neg	Neg
Unknown	Neg	Neg	Neg	Neg	Neg	Neg	Neg
Unknown	Neg	Neg	Neg	Neg	Neg	Neg	Neg
Unknown	Neg	Neg	Neg	Neg	Neg	Neg	Neg
Unknown	Neg	Neg	Neg	Neg	Neg	Neg	Neg
2	Neg	Neg	Neg	Neg	Pos	Neg	Neg
Unknown	Neg	Neg	Neg	Neg	Neg	Neg	Neg
Unknown	Neg	Neg	Neg	Neg	Neg/Eq	Neg	Neg
7	Neg	Neg	Neg	Neg	Pos	Neg	Neg
0	Neg	Neg	Neg	Neg	Pos	Neg	Neg
4	Neg	Neg	Neg	Neg	Eq	Neg	Neg
3	Neg	Neg	Neg	Neg	Neg/Eq	Neg	Neg
6	Neg	Neg	Neg	Neg	Neg/Eq	Neg	Neg
3	Neg	Neg	Neg	Neg	Neg/Eq	Neg	Neg
9	Neg	Neg	Neg	Neg	Pos	Neg	Neg
1	Neg	Neg	Neg	Neg	Neg	Neg	Neg
3	Neg	Neg	Neg	Neg	Pos	Neg	PZ
3	Neg	Neg	Neg	Neg	Neg/Eq	Neg	Neg
6	Neg	Neg	Neg	Neg	Neg	Neg	Neg
1	Neg	Neg	Neg	Neg	Neg	Neg	Neg
Unknown	Neg	Neg	Neg	Neg	Neg	Neg	Neg
Unknown	Neg	Neg	Neg	Neg	Neg	Neg	Neg
4	Neg	Neg	Neg	Neg	Neg/Eq	Neg	Neg
2	Neg	Neg	Neg	Neg	Pos	Neg	Neg
6	Neg	Neg	Neg	Neg	Neg	Neg	Neg

*dpo, days postsymptom onset; Eq, equivalent; MAC-ELISA, IgM-capture ELISA; Neg, negative; Pos, positive; PZ, possible Zika virus; RT-PCR, reverse transcription PCR.

Although specificity is a key factor, for a front-line diagnostic test, sensitivity is a major factor in determining its usefulness. With appropriate diagnostic testing in place, including use of Zika virus conformational cross-PRNTs, false-positive results caused by specificity issues can usually be overcome. However, poor sensitivity will lead to false-negative results that might not be followed up by testing of additional sample collections. When compared with the in-house diagnostics (MAC-ELISA with PRNT confirmation), the IgM assays of Euroimmun, Abcam, and Novatec demonstrated sensitivities of 37%, 57%, and 65%, respectively. When we combined results of the Euroimmun IgM and IgG ELISAs, sensitivity increased to 82%. The InBios Zika Virus Detect IgM assay correctly identified all confirmed Zika IgM-positive samples identified by the recommended diagnostic assays, resulting in a sensitivity of 100%. The InBios ELISA also detected IgM in 50% of samples that were positive for Zika virus by RT-PCR, whereas the other assays did not detect IgM in most of these samples (Table 3).

**Table 3:** Detection of IgM in RT-PCR–positive serum samples by using in-house and commercial Zika virus serologic assays*

Sample collection dpo	In-house Zika virus diagnostic results			DENV PRNT	Commercial Zika virus serologic assays results				
	RT-PCR	MAC-ELISA	PRNT		Euroimmun IgM	Euroimmun IgG	Novatec IgM	Abcam IgM	InBios IgM
0	Pos	Neg	ND	ND	Neg	Neg	Neg	Neg	Neg
2	Pos	Neg	ND	ND	Neg	Neg	Eq	Neg	Pos
5	Pos	Neg	ND	ND	Neg	Neg	Neg	Neg	Neg
7	Pos	Pos	ND	ND	Pos	Pos	Pos	Pos	Pos
3	Pos	Neg	ND	ND	Neg	Neg	Neg	Neg	Neg
2	Pos	Neg	ND	ND	Neg	Neg	Neg	Neg	Pos
2	Pos	Pos	ND	ND	Neg	Neg	Pos	Neg	Pos
3	Pos	Neg	ND	ND	Neg	Neg	Neg	Neg	Pos
3	Pos	Neg	ND	ND	Neg	Neg	Pos	Neg	PZ
2	Pos	Neg	ND	ND	Neg	Neg	Neg	Neg	Neg

*DENV, dengue virus; dpo, days postsymptom onset; Eq, equivalent; MAC-ELISA, IgM-capture ELISA; ND, not done; Neg, negative; Pos, positive; PRNT, plaque reduction neutralization test; PZ, possible Zika virus; RT-PCR, reverse transcription PCR.

## Conclusions

The low sensitivity of most immunoassays evaluated could be improved by testing a repeat sample collected a few weeks after the initial specimen, although this sampling is not always practical, particularly if resources are limited. When performed in combination, the Euroimmun Zika Virus IgM and IgG ELISAs provide improved sensitivity. However, interpretation of recent versus past infections could be problematic, particularly when IgM results are negative and IgG results are positive. On the basis of our findings, the InBios Zika Virus Detect MAC-ELISA provides diagnostic results comparable to the CDC-based in-house MAC-ELISA for specimens collected from patients with primary flavivirus exposures (i.e., no detectable background immunity to DENV). A needed follow-up to our study will be further evaluation of IgM detection by commercial ELISAs involving cases of secondary flavivirus exposures or previous immunization to related viruses, such as yellow fever virus.
